# Concerns and considerations among caregivers of a child with autism in Qatar

**DOI:** 10.1186/1756-0500-5-290

**Published:** 2012-07-06

**Authors:** Nadir M Kheir, Amy L Sandridge, Sara A Hayder, Muna S Al-Ismail, Fadhila Al-Rawi

**Affiliations:** 1 Qatar University, Doha, Qatar; 2 Saint Joseph College, West Hartford, CT, USA; 3 Research and Education Centre, Aspetar - Qatar Orthopaedic and Sports Medicine Hospital, Doha, Qatar; 4 Hamad Medical Corporation, Doha, Qatar

**Keywords:** Autism, Qatar, Caregivers, Pessimism, Rehabilitation services, Faith

## Abstract

**Background:**

Autism impacts the lives of the family looking after a child with the condition in different ways, and forces family members to modify their daily lives to suit their reality. To our knowledge, no previous research investigated concern and considerations of parents/caregivers of children with autism in Qatar or the Arabic speaking Middle Eastern region.

**Methods:**

Caregivers of a child who was between the age of 3 to17 years old at the time of the study and who was diagnosed with ASD (Autistic Group or AG) were recruited from the two main developmental pediatric and children rehabilitation clinics in Qatar. The control group (non-autism group, or NAG) was represented by caregivers of a non-autistic child between the age of 3 to 17 years old at the time of the study and who were visiting a family clinic of a primary health care facility for routine medical check-up. Data collected from both groups included related to the child (e.g. the child’s date of birth, his/her relation to the caregiver, number of siblings, number of hours of sleep in a day, number of hours spent watching television or videos prior to age 3, time spent indoors prior to age 3, absenteeism from school, and use of a nanny to care for the child) and to the caregiver (education level, profession, level of consanguinity using the phylogram method). In addition to these questions, caregivers in the AG were asked specific questions around maternal concern and considerations in respect to the future of their children and the specialized services they receive.

**Results:**

Children in the autism group spent more time indoors, watching television, or sleeping than children in the non-autism group. Only around 40% of caregivers in the autism group said they would encourage their child to get married and become a parent when s/he grows up. A number of caregivers of children with autism frequently utilize specialized rehabilitation services; others did express their needs for these services and made comments about having to wait a long time before they were provided with some of the services. Religious faith helped caregivers in accepting having a child with autism. General health-related quality of life did not differ significantly between the caregivers of the two groups, although mental health was consistently poorer in the autism group of caregivers.

**Conclusions:**

The study draws attention to the concerns of the families of children with autism and their expectations about the future of their children. The findings can be used by policy makers in planning services to support these families in Qatar.

## Background

Children with autism may present a clear psychological concern to their parents and caregivers. Factors such as lack of functional independence, severe maladaptive behaviors and severity of autism were strongly linked to poorer quality of life (QoL) [1]. In addition, physical health issues which are recently becoming recognized in the literature and the volume of hours spent looking after a child with autism can also contribute to caregiver burden [2-4]. There are also financial concerns associated with raising a child with autism which impacts on the society and families. A recent study showed that children with autism cost their families more than children with physical disability and three times as much as their typical peers [5]. Maternal pessimism was found to be associated with functional independence and severity of autism, the characteristics of the mother (lower levels of pessimism) were predictive of more positive mother–child relationships [6]. A plethora of psychosocial problems affecting caregivers of children with autism ranging from stress, depression, anxiety, restrictions of roles and activities, strain in marital relationships and diminished physical health were reported in the literature [7,8]. To our knowledge, no previous research has assessed the concerns and pessimism of parents/caregivers of children with autism in Qatar or the Arabic-speaking Middle Eastern region. There is undoubtedly a need to investigate how caring for a child with autism affects the lives of the parents/caregivers and to develop comprehensive health care strategies that not only target the persons diagnosed with autism, but also cares for and supports those who are in direct daily contact with someone with autism. This research project seeks to explore these relationships and to specifically assess the level of concern and pessimism of the primary caregiver of a child with autism in Qatar.

## Methodology

Ethical approval to conduct the study was obtained from the Medical Research Centre of Hamad Medical Corporation and Shafallah Medical Genetics Center. Caregivers of a child who was between the age of 3 to17 years old at the time of the study and who was diagnosed with autism using established screening and diagnostic tools (the Childhood Autism Rating Scale (CARS); the Autism Diagnostic Interview (ADI); the Autism Diagnostic Observation Schedule-General (ADOS-G); or the Diagnostic and Statistical Manual of Mental Disorders, Fourth Edition (DSM-IV)) were invited to participate in this cross-sectional exploratory study (Autism Group or AG) from the two main developmental pediatric and children rehabilitation clinics in Qatar [9,10]. Exclusion criteria were the presence of chronic medical conditions that might interfere with study, recent (<2 months) initiation of behavior therapy, and/or recent (<2 months) changes in the nuclear family’s life (marriage, divorce, death of a family member).

The non-autism group (NAG) was represented by caregivers of a typically developing child between the age of 3 to 17 years old at the time of the study and who were visiting a family clinic of a primary health care facility for routine medical check-up. Exclusion criteria in the NAG were the presence of a household child with autistic disorder, other neurodevelopental disorder, psychotic disorders, and/or recent (<2 months) changes in nuclear family’s life (marriage, divorce, death of a family member).

During a scheduled visit to the clinic, eligible participants from both groups (i.e. AG and NAG) were provided with information about the study and asked if they would participate. Those who agreed, signed informed consent. Demographic and information related to the child’s life at home were collected from both groups and included information related to the child (e.g. the child’s date of birth, his/her relation to the caregiver, number of siblings, number of hours of sleep in a day, number of hours spent watching television or videos prior to age 3, time spent indoors prior to age 3, absenteeism from school, and use of a nanny to care for the child) and to the caregiver (education level, profession, level of consanguinity using the phylogram method) [11]. In addition to these questions, caregivers in the AG were asked specific questions relating to maternal concern and considerations related to the future of their children and the specialized services they receive. These questions included whether the caregiver will encourage the child to marry or have children and how accepting the caregiver was of having a child with autism. They also included a question about whether religious faith helped the caregiver accept having a child with autism, and a set of questions related to the specialized services provided by the health care system or that the caregiver wished to receive were asked. The questions were developed utilizing information about maternal concern in the published literature and a consultative process and meetings that included members of the research team with pediatricians specializing in neurodevelopmental disorders, and other healthcare providers who work closely with families with children diagnosed with autism [12].

Quality of life was assessed using the Lebanese Arabic version of the Standard Recall SF-36v2 obtained through license from QualityMetric Incorporated. The SF-36v2 was interviewer-administered to all participants to protect against bias associated with issues of literacy. Quality of life data manipulation, entering, and scoring followed instructions provided in the SF-36v2 Health Survey: A Primer for Health Care Providers.

Data were managed according to standard epidemiological practice. All data were analyzed using the Statistical Package for Social Sciences (SPSS®) version 18.

Chi-Square was used to assess change between group variables (categorical data) and the Student’s *t*-test assessed differences between group variables (numerical data). Statistical significance was set at p value of <0.05. Pearson correlation coefficient was used to measure the association between QoL variables and patient demographics.

## Results

### Sample characteristics

From a total of 100 caregivers eligible for inclusion in the AG group and invited to participate in the study, 56 (56%) gave consent and ultimately provided data. Forty eight caregivers of a neurotypically-growing child (NAG) from a total of 100 approached participated (48%). The remaining 44 AG children and 52 NAG children were unable to be contacted despite numerous attempts. Given the high mobility of families in Qatar this response rate is not alarming. No statistically significant difference in the education level was found between the two groups of caregivers (p > 0.05).

As illustrated in Table 1, the majority of the respondents in both groups of caregivers held professional jobs or were homemakers (or retirees) (p > 0.05).

**Table 1:** Comparison between the educational levels and professions of the study groups (n = 98)

Category	Autistic Group (n = 56) N (%)	Non Autistic Group (n = 42) N (%)
**Caregiver Educational level**		
Primary	1 (2)	1 (2)
Preparatory	1 (2)	2 (4)
Secondary	12 (21)	12 (29)
Some University	17 (30)	10 (24)
Bachelor degree	21 (38)	15 (36)
More than Bachelor degree	4 (7)	2 (5)
**Caregiver Profession**		
Administrative	11 (20)	8 (19)
Technical	2 (4)	7 (17)
Professional	23 (41)	15 (36)
Homemaker/Retired	16 (28)	9 (21)
Unknown	4 (7)	3 (7)
**Caregiver’s Spouses’ Profession**		
Administrative	13 (23)	11 (26)
Technical	1 (2)	1 (2)
Professional	23 (41)	20 (48)
Homemaker/Retired	15 (27)	3 (7)
Unknown	4 (7)	7 (17)

While most of the spouses in the two groups were not consanguineously related, about 8% of the AG and 3% of the spouses in NAG were double-first cousins (where two siblings marry two siblings). The number of siblings of the selected child of the parent/caregiver in each group is listed in Table 2. AG children had more siblings than NAG children (average of 2.6 for AG and 2.2 for NAG, p = 0.002) but looking at the full distribution we see that a higher percentage of NAG children had 2 or 3 siblings.

**Table 2:** Number of full siblings in the two groups (n = 98)

Count	Autistic Group (n = 56) N (%)	Non Autistic Group (n = 42) N (%)
0	5 (9)	3 (7)
1	13 (23)	6 (14)
2	11 (20)	14 (33)
3	15 (27)	17 (41)
4	5 (9)	2 (5)
5	2 (4)	0
6	0	0
7	3 (5)	0
8	2 (4)	0

The number of mothers interviewed was more than twice the number of fathers (64 to 27; p < 0.05). In the AG, 23 fathers were interviewed (mostly caretakers of their sons) while in the NAG only 4 fathers were interviewed about their sons.

In the AG group, data were missing for sex of the caregiver in 2 cases and the sex of the child in 1 case. In the NAG group there were 4 children for whom the sex of the caregiver was not collected.

The majority of children in the two groups had no other health conditions (77% and 80% of the AG and NAG, respectively). However, around 70% of the children in the AG were prescribed other medications for a variety of acute and minor conditions (e.g., influenza, ear infection, gastrointestinal problems). 

The time that the child spent indoors, watching television or videos before age 3, and sleeping is presented in Table 3. In the case where the child did the activity “all day” this was coded to the maximum value of 22 h for time spent indoors and 16 h for the television and video watching hours.

**Table 3:** Summary statistics for time spent by children indoors, watching TV and sleeping, by sex

	Group	Mean (SD)	Median (Range)	P value^1^	P value^2^
Before the age of 3, how many hours a day did the child spent indoors?	**Autism (n = 53)**	17.1 (5.9)	20.0 (6–22)	<0.001	
	Male (n = 22)	17.3 (6.2)	22.0 (6–22)		0.013
	Female (n = 31)	17.0 (5.7)	20.0 (6–22)		<0.001
	**Non-Autism (n = 41)**	10.3 (4.2)	10.0 (3.5–22)		
	Male (n = 7)	10.7 (3.5)	10.0 (6–16)		
	Female (n = 34)	10.2 (4.3)	10.0 (3.5–22)		
Before the age of 3, how many hours a day did the child watch television?	**Autism (n = 52)**	4.7 (4.1)	4.0 (0–16)	0.015	0.420
	Male (n = 21)	4.0 (3.9)	3.0 (0–15)		
	Female (n = 31)	5.2 (4.3)	4.0 (0–16)		0.020
	**Non-Autism (n = 42)**	2.9 (2.6)	2.0 (0–13)		
	Male (n = 8)	2.8 (2.4)	2.0 (0–7)		
	Female (n = 34)	2.9 (2.7)	2.0 (0–13)		
Before the age of 3, how many hours a day did the child sleep?	**Autism (n = 52)**	8.4 (1.9)	8.0 (4–16)	0.001	
	Male (n = 22)	7.6 (1.5)	8.0 (4–10)		0.001
	Female (n = 30)	8.9 (2.0)	8.5 (6–16)		0.19
	**Non-Autism (n = 42)**	9.5 (1.3)	9.5 (7–12)		
	Male (n = 8)	9.9 (1.3)	10.0 (8–12)		
	Female (n = 34)	9.4 (1.3)	9.5 (7–12)		

P value^1^ compares mean of entire Autism and Non-Autism groups

P value^2^ compares the Autism females to the Non-Autism females and the Autism males to the Non-Autism males

The longest time a boy in the NAG spent indoors before the age of 3, on average, was 16 h. Two girls in this group were reported to spend on average 22 h a day indoors before the age of 3.

When the data were stratified by sex we see that the mean number of hours spent watching television before the age of 3 for the AG boys is not significantly different from the mean number of hours of the NAG group. However, girls in the AG spent more time watching television before the age of 3 than girls in the NAG (p ≤ 0.05). The AG boys have a statistically significant lower number of sleeping hours before the age of 3 than the NAG boys but for the girls there is no statistical difference.

When asked if they would encourage their child to get married when s/he grows up, 40% of the caregivers in the AG said they would and 20% said they would not (p < 0.05). The remaining 40% said they did not know if they would encourage this child to ever get married. Thirty eight percent of the caregivers in the AG would encourage their child to become a parent when the child gets older, but 18% said they would not (p < 0.05). The remaining caregivers did not know if they would encourage their child to have children of their own.

Around 45% of the caregivers said they ‘now’ accept having a child with autism compared to around 9% who do not accept this reality (p < 0.05). However, over 45% of the caregivers refused to answer this question. When the child was first diagnosed, around 20% did not accept the idea of having a child with autism compared to around 22% who still do not accept this idea. Again, the remaining caregivers did not wish to answer this question. When asked if their religious faith helped them cope with the challenges of having a child with autism, around 50% agreed that it did, and 48% declined to answer this question.

Only 30 of the 56 caregivers in the AG (54%) responded to the service questions. Of these, 57% indicated that special education classes are available for their child, and the remaining said that these were not available and they desired these classes for other caregivers (p < 0.05). Around 50% of parents said that (as caregivers) they have access to parent support groups to help them cope, but 50% said they do not have access to such services and that they wished that they did have access. A small minority (3%) said there was no need for special classes for caregivers of children with autism.

In respect to QoL, there appears to be no statistically significant difference between QoL domains of the two groups of caregivers, but caregivers in the AG rated their health as poor and likely to get worse (p < 0.05). An analysis of the Physical Component Summary and the Mental Component Summary (PCS and MCS, respectively) scores of the Short Form-36v2 revealed an overall poorer mental health in the AG compared to the NAG although neither reached statistical significance (p > 0.05). The sex of the parent/caregiver appears to be associated with several quality of life domains. Female caregivers suffered more bodily pain than men (p = 0.000). They also had more fatigue and tiredness (low vitality) than men (p = 0.003). They had more problems with work or other usual activities as a result of emotional problems (p = 0.039). Mental Component Summary (MCS) scores indicated significantly poorer mental health among the females in this study compared to males (p < 0.05).

## Discussion

This study provides a snapshot of the concerns the caregivers of children with autism in Qatar harbor regarding their child’s life, future, and care. Studies conducted elsewhere suggested that parents of children with autism seem to experience more stress than parents of typically developing children, and that maternal pessimism was associated with functional independence and severity of autism [12,13]. The degree of social impairment of the child and the severity of his/her condition are believed to be associated with the caregiver’s burden and concern [12]. Our cohort of children in the AG spent more time indoors, watching television or sleeping before the age of 3 than children in the NAG, showing features of social deprivation, isolation, and low functional ability. The long time a child spends indoor in this group could additionally reflect a tendency among caregivers of children with autism to keep their children isolated from the outside world. This could be due to a perceived lack of social and community understanding of the nature of autism and may mirror the stigma attached with this condition in the mind of the average person in this region. People may feel helpless and awkward when they find themselves in a situation where they have to deal with a child with autism or with his/her family, and this feeling may be intuited by the caregivers of the children, adding to their psychosocial burden.

Although it was assumed that the average time indoors until the age of 3 might be greater for girls with autism, an effect of gender on time spent indoors has not been demonstrated in this study. Whether greater time indoors after the age of 3 as compared to neurotypical children continues to be shown as children age will have to be investigated in future studies. The amount of time spent indoors prior to the age of 3 could be an interesting risk factor to explore further [14]. With respect to the watching of television, studies conducted elsewhere reported similar findings to ours. For example, Chonchaiya and colleagues found that children with autism spectrum disorders began to watch television significantly earlier than controls and spent more time watching television than typically developing children [15]. Since television is nearly always watched indoors, separating out the affect of being indoors versus watching television in and of itself would have to be considered. And as Larsson et al., (2009) demonstrated, the issues may be exposure to maternal smoking, low ventilation in the home and polyvinyl chloride (PVC) flooring which this study did not consider [14]. Interestingly, a study published in 2011 reported that adolescents with autism spectrum disorders spent considerable time in discretionary activities, with watching television and using a computer as the most frequent activities. The children in their study also spent little time engaged in conversations or doing activities with peers. This behavior could be perceived as a predictor for future development of the child. Indeed, it had been suggested that greater time spent in conversation and reading predicted future decreases in severity of social impairment, and the way that children and adolescents with autism spend their free time may have implications for their development and the course of their autism symptoms [16].

Questions related to the caregiver’s predictions about the future of their child (getting married or having children) were used as indicators of the caregiver’s concerns. Around half of the AG caregivers did not wish to answer questions about whether they would encourage their children to get married or become parents when they grow up. This might be a reflection of the innate concern and pessimism these caregivers experience in respect to the future of their children. Refusing to answer these set of questions could also be associated with parental concern and stress. We had more fathers in the AG than mothers, and studies have shown that concern of fathers of children with intellectual disabilities was associated with the experienced social acceptance of the child, whereas in mothers it was the child’s behavioral problems [17]. This could explain why so many respondents refused to answer these specific questions, all of which had social implications. Another explanation could be cultural, in that speculation on what will happen in a child’s life questions the tenent of one’s life being in God’s (Allah’s) hands. However, the true reasons of this phenomenon remain speculative. This is a limitation of our study and might be addressed through focus groups in the future. Faith did help a number of the caregivers of children with autism in coping. In an Islamic society like the one in Qatar, this should not be a surprising finding. Islamic teaching emphasizes virtues of endurance, resilience and acceptance of ill health, just like good health and good fortune, as both are the will of God and could be to test one’s faith and belief according to Islamic teachings. Indeed, studies on families of children with autism reported that parents coped through their religious faith and other emotion-focused strategies [18,19].

Interestingly, the number of fathers who answered our questionnaires as the principal caregivers of their children with autism far exceeded the number of mothers in our AG in this study. This is a somewhat unexpected finding that suggests greater involvement of fathers in the care of their children in an area that has traditionally been dominated by mothers. However, another explanation is that given the societal penchant in the Middle East for men to be in the public sphere more than women, it could be that more fathers in our sample took the AG child to the clinic than would be expected and presented as the primary caretaker. Additionally, within the Middle Eastern culture, often the male is considered head of household and tends to speak on behalf of the family.

While AG had slightly more siblings overall than NAG siblings (Table 2), interesting suggestions can be made from the comparison of the two groups. Nine percent of the AG had no siblings as compared to 7% of the NAG. Twenty-three percent of the AG only had one sibling as compared to 14% of the NAG. At 2 siblings, the trend reverses with 20% of the AG having 2 siblings and 33% of the NAG having 2 siblings. In the AG group, only 27% had 3 siblings but 40% had 3 siblings in the NAG group. This could indicate that, in some families, a decision is made to limit family size based on the fact that there is an autistic child in the family who needs care although this conclusion would require further study.

While a number of caregivers in the AG utilize specialized services, like special education classes and parent support groups, others said these services were unavailable to them. Other studies overseas suggested that participation in such services reflects some degree of perceived benefit, especially given the competing time and resource demands of caring for a child with autism [20]. They also concluded that support group participants are more likely to be middle income and well educated individuals. Qatar has made tremendous efforts towards improving and upgrading the available services in the last few years. The two clinics from which participants in the study were recruited provide a number of free services, which include parent support group classes, speech therapy, music therapy, field trips, sibling day, and at least one of the two clinics is actively planning for an adaptive physical education class to integrate sport in the comprehensive care package for the the children. However, the expressed need for special services among a subset of participants in this study reflects some genuine caregivers’ concern about challenges facing the service providers in Qatar at the time of writing this manuscript, and possibly also reflects a degree of ignorance among caregivers about the availability of some of these services . Based on informal communications and interactions with staff, clinicians and families of children with autism during the course of this project, some of the challenges include long waiting lists, inconsistent provision of services, inadequacy in respect to staff numbers and expertise, complicated processes of referral, and sometime language barriers between the service providers and service beneficiaries.

Most of the QoL domains of caregivers of children with autism reflected poorer mental health compared to caregivers of neurotypical children, although the differences were not reaching statistical significance in most of the domains compared. Only the General Health domain was significantly poorer in the AG caregivers. This is the domain that reflects how respondents rate their health and how they estimate it to become in future. It was clear that caregivers of children with autism rated their general wellbeing as poor and they were expecting it to get worse.

There is so far no QoL instrument specifically designed for use with caregivers of developmental disorders, including autism. Most of the instruments available were constructed as measures for caregiver’s burden or strain, and only a few of them are autism-specific instruments [21,22]. It is important to make a distinction between these attributes of health and well-being; where burden and strain are conceptually different from, and not necessarily synonymous with, health-related QoL. Indeed, it had been articulated in the literature that the QoL of caregivers could be improved even with the pressures in their lives. It has been suggested that the focus in caregiving research on burden should be supplemented with an emphasis on QoL [23].

The results of our study may suffer from information bias. The persons who reported that they were the primary caregiver (usually the biological mother or the father) may not have been the actual day-to-day caregiver. We allowed the caregiver to self-identify as we could not ask the child with autism. Hence, realistically the actual primary caregiver of the person with autism in this population could have been the mother, the father, or the nanny or housemaid. Housemaids are easily employed in Qatar through employment agencies. However these caregivers are usually from a low socioeconomic class and with low literacy skills; thus not especially trained to work with people with special needs. Another limitation was the large number of caregivers in the AG who refused to answer questions relating to their predictions of their child’s future, their acceptance of having a child with autism, and the role of faith in helping them cope. While this is obviously an important limitation, it does reflect a degree of innate concern and pessimism, thus providing further proof to the degree of anxiety, concern, and pessimism these caregivers might be feeling.

One of the major limitations of our work was the poor response rate. Only 56% of the 100 families with autistic children were able to be contacted and interviewed. For the controls it was only 48%. This would likely introduce response bias. However, with respect to the AG families, mobility in Qatar is so frequent and spans socio-economic strata that we may have in fact captured a random selection of families with autistic children. With respect to the NAG, the research assistants (students) systematically collected the data, but were unable to contact all the 100 families due to academic responsibilities. Therefore, the findings from this study should be treated cautiously.

## Conclusions

The findings of this study suggest innate concerns harbored by caregivers of children with autism and translated into pessimism about the future of the child in respect to living a socially normal life (marriage, having children). Children with autism also spent more time indoors before the age of 3 than neurotypically developing children, which could be predictor for greater social impairment in the future. The study revealed areas relating to concerns about support provided to children with autism and their caregivers and the status of these children in different aspects. The findings should help health policy-makers provide better and more focused supports to the children with autism and their families.

## Availability of supporting data

The data set supporting the results of this article is available in the [ComparisonsAllDataFINAL-AS-19Sep10e] repository, [http://dl.dropbox.com/u/7353852/ComparisonAllDataFINAL-AS-19Sep10.xls].”

## Competing interests

No competing interest to declare.

## Authors’ contributions

NMK: Methodology, QoL data management, supervised data collection, supervised data entry, wrote the manuscript, corresponding author. OMG: Methodology, supervised data collection, supervised data entry, revised manuscript. ALS: Methodology, questionnaire development, supervised data entry and validation, data analysis, revised manuscript. SAH: Sample recruitment, data collection, data entry and validation, revised manuscript. MSA: Sample recruitment, data collection, data entry and validation, revised manuscript. FAR: Sample logistics and recruitment, revised manuscript. All authors read and approved the final manuscript.

## References

[B1] McSweeny AJ Creer TL Health-related quality-of-life assessment in medical care Dis Mon 1995 41 1 71 7805548

[B2] Durkin MS Maenner MJ Meaney FJ Levy SE DiGuiseppi C Nicholas JS Socioeconomic inequality in the prevalence of autism spectrum disorder: evidence from a U.S. cross-sectional study PLoS One 2010 5 e11551 10.1371/journal.pone.0011551 20634960 PMC2902521

[B3] Fernell E Gillberg C Autism spectrum disorder diagnoses in Stockholm preschoolers Res Dev Disabil 2010 31 680 685 10.1016/j.ridd.2010.01.007 20149593

[B4] Foley NM Doobay AF Assouline SG Parent, teacher, and self perceptions of psychosocial functioning in intellectually gifted children and adolescents with autism spectrum disorder J Autism Dev Disord 2010 40 1028 1038 10.1007/s10803-010-0952-8 20143145

[B5] Xiong N Yang L Yu Y Hou J Li J Li Y Investigation of raising burden of children with autism, physical disability and mental disability in China Res Dev Disabil 2011 32 306 311 10.1016/j.ridd.2010.10.003 21055902

[B6] Orsmond GI Seltzer MM Greenberg JS Krauss MW Mother–child relationship quality among adolescents and adults with autism Am J Ment Retard 2006 111 121 137 10.1352/0895-8017(2006)111[121:MRQAAA]2.0.CO;2 16466284

[B7] Holmes N Carr J The pattern of care in families of adults with a mental handicap: a comparison between families of autistic adults and Down syndrome adults J Autism Dev Disord 1991 21 159 176 10.1007/BF02284757 1830876

[B8] Shu BC Quality of life of family caregivers of children with autism: the mother’s perspective Autism 2009 13 81 91 10.1177/1362361307098517 19176578

[B9] American Psychiatric Association Diagnostic and statistical manual of mental disorders 1994 4 American Psychiatric Association, Washington DC Ref Type: Report

[B10] Rellini E Tortolani D Trillo S Carbone S Montecchi F Childhood Autism Rating Scale (CARS) and Autism Behavior Checklist (ABC) correspondence and conflicts with DSM-IV criteria in diagnosis of autism J Autism Dev Disord 2004 34 703 708 10.1007/s10803-004-5290-2 15679189

[B11] Sandridge AL Takeddin J Al-Kaabi E Frances Y Consanguinity in Qatar: knowledge, attitude and practice in a population born between 1946 and 1991 J Biosoc Sci 2010 42 59 82 10.1017/S002193200999023X 19895726

[B12] Lin LY Factors associated with caregiving burden and maternal pessimism in mothers of adolescents with an autism spectrum disorder in Taiwan Occup Ther Int 2011 18 96 105 10.1002/oti.305 20925134

[B13] Orsmond GI Seltzer MM Adolescent siblings of individuals with an autism spectrum disorder: testing a diathesis-stress model of sibling well-being J Autism Dev Disord 2009 39 1053 1065 10.1007/s10803-009-0722-7 19291379 PMC2826848

[B14] Larsson M Weiss B Janson S Sundell J Bornehag CG Associations between indoor environmental factors and parental-reported autistic spectrum disorders in children 6–8 years of age NeuroToxicology 2009 30 822 831 10.1016/j.neuro.2009.01.011 19822263 PMC3787697

[B15] Chonchaiya W Nuntnarumit P Pruksananonda C Comparison of television viewing between children with autism spectrum disorder and controls Acta Paediatr 2011 100 1033 1037 10.1111/j.1651-2227.2011.02166.x 21244489

[B16] Orsmond GI Kuo HY The daily lives of adolescents with an autism spectrum disorder: discretionary time use and activity partners Autism 2011 15 579 599 10.1177/1362361310386503 21697194 PMC3572828

[B17] Saloviita T Italinna M Leinonen E Explaining the parental stress of fathers and mothers caring for a child with intellectual disability: a Double ABCX model J Intellect Disabil Res 2003 47 300 312 10.1046/j.1365-2788.2003.00492.x 12787162

[B18] Gray DE Coping over time: the parents of children with autism J Intellect Disabil Res 2006 50 970 976 10.1111/j.1365-2788.2006.00933.x 17100957

[B19] Gray DE Accommodation, resistance and transcendence: three narratives of autism Soc Sci Med 2001 53 1247 1257 10.1016/S0277-9536(00)00424-X 11556614

[B20] Mandell DS Ittenbach RF Levy SE Pinto-Martin JA Disparities in diagnoses received prior to a diagnosis of autism spectrum disorder J Autism Dev Disord 2007 37 1795 1802 10.1007/s10803-006-0314-8 17160456 PMC2861330

[B21] Hirschman KB Shea JA Xie SX Karlawish JH The development of a rapid screen for caregiver burden J Am Geriatr Soc 2004 52 1724 1729 10.1111/j.1532-5415.2004.52468.x 15450052

[B22] Khanna R Madhavan SS Smith MJ Tworek C Patrick JH Becker-Cottrill B Psychometric properties of the Caregiver Strain Questionnaire (CGSQ) among caregivers of children with autism Autism 2011 2 179 199 21715548 10.1177/1362361311406143

[B23] Chappell NL Reid RC Burden and well-being among caregivers: examining the distinction Gerontologist 2002 42 772 780 10.1093/geront/42.6.772 12451158

